# Improvements in Optical Characteristics after Excision of an Overhanging Bleb Developed following Trabeculectomy

**DOI:** 10.1155/2021/7433987

**Published:** 2021-09-04

**Authors:** Yu Mizuno, Atsushi Hirota, Kazuyuki Hirooka, Yoshiaki Kiuchi

**Affiliations:** ^1^Department of Ophthalmology and Visual Science, Hiroshima University, 1-2-3 Kasumi Minamiku, Hiroshima 734-8551, Japan; ^2^Hirota Eye Clinic, 1-25-1 Shinmachi Shyunanshi, Yamaguchi 745-0017, Japan

## Abstract

**Introduction:**

To examine the corneal total higher-order aberrations before and after the excision of an overhanging bleb that developed following trabeculectomy. *Case Presentation*. Two patients who developed overhanging blebs following trabeculectomy with a fornix-based conjunctival flap using mitomycin C (MMC) were assessed. We measured the corneal total higher-order aberrations for a 4 mm pupil diameter using the TOPCON KR-1W wavefront analyzer and the visual acuity before and after bleb excision. The corneal total higher-order aberration (HOA) improved from 0.50 *μ*m to 0.38 *μ*m in case 1 and from 0.59 *μ*m to 0.49 *μ*m in case 2 after bleb excision. The intraocular pressure was identical before and after bleb excision in both cases. No significant changes in the best-corrected visual acuity (BCVA) were noted in case 1; however, BCVA was improved from 20/25 to 20/20 in case 2. Both cases showed improvements in the symptoms of dysesthesia.

**Conclusion:**

Excision of the overhanging bleb developed following trabeculectomy may have beneficial possibility in some cases where corneal total HOA is affected and reduces the symptoms of dysesthesia.

## 1. Introduction

Glaucoma is an optic neuropathy characterized by gradual progressive morphological changes in the optic disc that leads to visual field loss [1]. Trabeculectomy is an effective surgery that is performed for lowering the intraocular pressure (IOP) in patients with glaucoma to hinder the progression of visual field loss. However, following this surgery, patients occasionally complain of foreign body sensation, poor cosmetic appearance, and reduction in vision. Several studies have shown that trabeculectomy results in changes in the corneal keratometry, astigmatism, and topography, which lead to change in vision [2, 3]. In such patients, some have overhanging blebs (OHBs), which are defined as oversized filtering blebs that cover part of the cornea. Recently, advances in ocular aberrometry have revealed that ocular surgeries increase ocular and corneal higher-order aberrations (HOAs). Here, we report cases of two patients who developed OHB following trabeculectomy using mitomycin C (MMC). We examined the optical characteristics before and after excision of OHBs by a wavefront analyzer, which can objectively simulate the retinal image of Landolt C using its simulation system for the first time.

## 2. Higher-Order Aberration Measurement and Surgical Procedures

**Figure 1:**
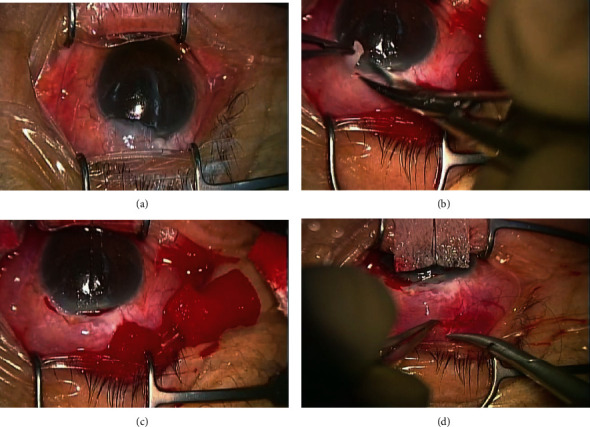
Intraoperative photographs of the excision of the overhanging bleb (OHB). The yellow arrows are the range of the OHB. The OHB is avascular and thin walled (a). A portion of the OHB, which is easy to remove from the cornea, is excised immediately anterior to the limbus using scissors (b). Several sponges soaked in 0.04% MMC solution are applied for 5 min in the scleral surgical site (c). The incision were sutured using a 10-0 nylon suture (d).

The corneal total higher-order aberrations for a 4 mm pupil diameter without dilating pupil were measured before and after bleb excision to examine the effects of the excision of the OHB using the TOPCON KR-1W wavefront analyzer (Topcon Corporation, Tokyo, Japan). The wavefront aberrometer was a device for measuring the wavefront aberrations of the eye; the retinal image of Landolt C was objectively simulated using its simulation system. The retinal image resulted from the convolution of the paraxial image and the point-spread function [4]. It could also be calculated by considering only the effect of the anterior cornea. The surgical technique of OHB excision was followed. The overhanging portion of the bleb was removed from the cornea by excising it immediately anterior to the limbus using scissors. Following the dissection, a conjunctival incision was made 7–8 mm posterior to the limbus. An elongated crescent knife was inserted into the conjunctival edge to peel the strong adhesion in order to allow the aqueous humor drain to the fornix side of the eye, preventing the recurrence of OHB. Subsequently, though there was possibility to become avascular bleb, we inserted several sponges soaked in 0.04% MMC solution for 5 min application on the scleral surgical site to prevent strong fibrosis of the conjunctiva and sclera. This area was then washed with 100 ml of balanced salt solution. Thereafter, the incision was sutured with a 10-0 nylon suture (Figure 1).

## 3. Case Presentation

### 3.1. Case 1

**Figure 2:**
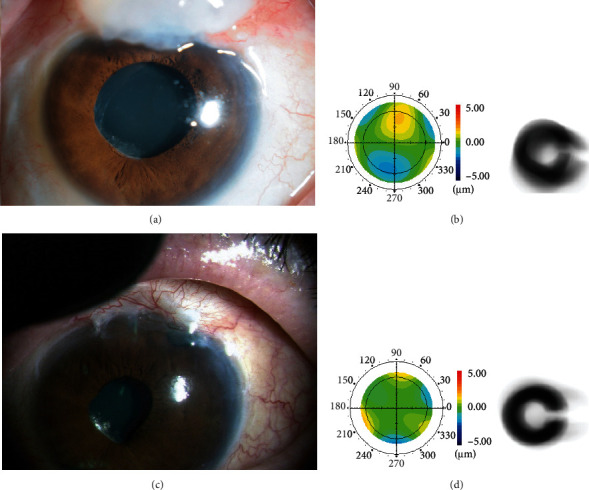
Slit-lamp examination of the OHB in case 1 (a). Excision of the OHB (c). Corneal total higher-order aberrations before (b) and after bleb excision (d) with the simulated retinal images of Landolt C from corneal total higher-order aberrations on the right side.

A 56-year-old man presented with visual disturbances, discomfort, and poor cosmetics in his left eye. He has been undergoing treatment for primary open-angle glaucoma (POAG) when he was 32 years old. Before visiting our hospital, he had undergone trabeculectomy for the first time when he was 45 years old and underwent second surgery of trabeculectomy at age 46 in his left eye using a fornix-based conjunctival flap with MMC. A slit-lamp examination of the left eye revealed a large OHB; the anterior chamber has a normal depth and the lens status was IOL (intraocular lens) (Figure 2(a)). Fundus examination revealed the cup disc ratio of 1.0 in the left eye. The best-corrected visual acuity (BCVA) was 20/125 in the right eye and 20/60 in the left eye. IOP was 11 mmHg in the right eye and 9 mmHg in the left eye without the use of antiglaucoma drugs. We resected the OHB in his left eye. There was a slight leakage around the fornix margin of the excision, but it stopped naturally on postoperative day 5 (Figure 2(c)). At 2 months after the operation, the residual bleb was diffused, there was no leakage, the BCVA was similar to that before the excision, and the IOP of the left eye remained at around 10 mmHg. The corneal total HOAs improved from 0.50 *μ*m to 0.38 *μ*m. Simulated retinal images of Landolt C based on corneal wavefront data had improved clearly with reduced total corneal HOAs. The symptom of constant foreign body sensation was also reduced, and improvement of poor cosmetic was also seen (Figures 2(b) and 2(c)).

### 3.2. Case 2

A 79-year-old woman presented with discomfort, poor cosmetics, and reduced vision in her right eye. She has been undergoing treatment for POAG since she was 59 years old. She had undergone trabeculectomy using a fornix-based conjunctival flap with 0.04% MMC for the first time when she was 60 years old in the right eye. However, IOP was increased gradually and she underwent second surgery of trabeculectomy at age 61, a third at age 75, and a fourth at age 77.

**Figure 3:**
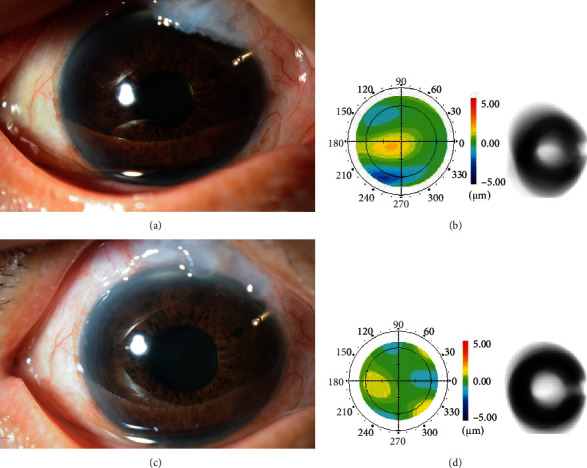
Slit-lamp examination of the OHB before (a) and after bleb excision (c) in case 2. Corneal total higher-order aberrations before (b) and after bleb excision (d) with the simulated retinal images of Landolt C from corneal total higher-order aberrations on the right side.

A slit-lamp examination of the right eye revealed an OHB; the anterior chamber has a normal depth and the lens status was IOL (intraocular lens) (Figure 3(a)). Fundus examination revealed the cup disc ratio of 1.0 in the right eye. The BCVA was 20/25 in the right eye, and she could discern the hand motion with her left eye. The IOP, without the use of antiglaucoma drugs, was 12 mmHg and 16 mmHg in the right eye and left eye, respectively. The patient underwent OHB excision in the right eye. No leakage was observed around the margin of the excision following the operation (Figure 3(c)). At 4 months after the operation, the residual bleb was diffused without any leakage and the right eye showed good IOP (10 mmHg). The corneal total HOAs improved from 0.59 *μ*m to 0.49 *μ*m; coma aberrations and spherical aberration also improved from 0.21 *μ*m to 0.12 *μ*m and from 0.19 *μ*m to 0.05 *μ*m, respectively. Simulated retinal images of Landolt C based on corneal wavefront data had clearly improved with reduced total corneal HOAs (Figures 3(b) and 3(d)). The BCVA was improved from 20/25 to 20/20 at 1 year after bleb excision. The symptoms of constant foreign body sensation and poor cosmetics were also reduced. Written informed consent was obtained from both patients to publish these findings and images.

## 4. Discussion

OHBs are a late complication following glaucoma filtration surgery, and the incidence of these blebs appears to be increasing with the use of antimetabolites [5]. Antimetabolites aid the antiproliferation of fibroblast cells, thereby preventing excessive postoperative scarring and enhancing the growth of the large bleb [6]. The mechanism underlying the formation of OHBs is complex. Several factors, such as the action of gravity on the OHB, the action of the eyelid, scar hyperplasia, and excessive aqueous overfiltration, may together contribute to the formation of OHBs. Following trabeculectomy, patients sometimes complain of a change not only in vision but also of dysesthesia and poor cosmesis. For patients with OHBs, treatment for extensive bleb migration onto the corneal surface requires surgical excision for long-term improvement in vision and symptoms. However, bleb leak that required suture repair may occur for the surgical excision [7]. The mechanism that results in the change of vision has yet not been fully understood. Recent advances in wavefront analysis have shown that ocular surgeries, such as trabeculectomy, corneal transplantation, and pterygium surgery, increase ocular and corneal HOAs, which affect the postsurgical visual function [3, 8, 9]. These are the first cases wherein OHB excision objectively improved comfort, poor cosmetics, and visual function by the wavefront aberrations without causing any significant change in IOP. Improvement in the corneal HOAs and the disappearance of the bleb on the cornea supported their subjective symptoms. BCVA did not improve in case 1 following the operation probably because of severe glaucoma and scotoma near the fixation points. Further clinical research by collecting data on OHB removal, wavefront analyzer, and visual outcome is needed to produce a definitive answer in the future. Our results should be compared with the results of data on those without OHB removal, so further research by collecting those data was also needed.

## 5. Conclusion

In conclusion, OHB excision may have beneficial possibility in some cases where corneal total HOAs are affected. Improvement of corneal total HOAs results in better quality of vision.
